# Molecular Evidence Shows Low Species Diversity of Coral-Associated Hydroids in *Acropora* Corals

**DOI:** 10.1371/journal.pone.0050130

**Published:** 2012-11-29

**Authors:** Silvia Fontana, Shashank Keshavmurthy, Hernyi Justin Hsieh, Vianney Denis, Chao-Yang Kuo, Chia-Ming Hsu, Julia K. L. Leung, Wan-Sen Tsai, Carden C. Wallace, Chaolun Allen Chen

**Affiliations:** 1 Biodiversity Research Center, Academia Sinica, Nangang, Taipei, Taiwan; 2 International Taiwan Graduate Program (TIGP)-Biodiversity, Academia Sinica, Nangang, Taipei, Taiwan; 3 Penghu Marine Biological Research Center, Makong, Penghu County, Taiwan; 4 Institute of Oceanography, National Taiwan University, Taipei, Taiwan; 5 Museum of Tropical Queensland, Townsville, Australia; The Australian National University, Australia

## Abstract

A novel symbiosis between scleractinians and hydroids (*Zanclea* spp.) was recently discovered using taxonomic approaches for hydroid species identification. In this study, we address the question whether this is a species-specific symbiosis or a cosmopolitan association between *Zanclea* and its coral hosts. Three molecular markers, including mitochondrial 16S and nuclear 28S ribosomal genes, and internal transcribed spacer (ITS), were utilized to examine the existence of *Zanclea* species from 14 *Acropora* species and 4 other Acroporidae genera including 142 coral samples collected from reefs in Kenting and the Penghu Islands, Taiwan, Togian Island, Indonesia, and Osprey Reef and Orpheus Island on the Great Barrier Reef, Australia. Molecular phylogenetic analyses of the 16S and 28S genes showed that *Acropora*-associated *Zanclea* was monophyletic, but the genus *Zanclea* was not. Analysis of the ITS, and 16S and 28S genes showed either identical or extremely low genetic diversity (with mean pairwise distances of 0.009 and 0.006 base substitutions per site for the 16S and 28S genes, respectively) among *Zanclea* spp. collected from diverse *Acropora* hosts in different geographic locations, suggesting that a cosmopolitan and probably genus-specific association occurs between *Zanclea* hydroids and their coral hosts.

## Introduction

Hydroids (Hydrozoa, Hydroida) establish relationships with a wide range of marine organisms, including anthozoans [1], [2], due to their ability to associate with various types of substrates, including substrate generalist species and species that live on the surface of other organisms such as algae, bryozoans, bivalves, and corals [3], [4]. These relationships range from epibiotic associations to stricter symbioses (mutualism or parasitism) [5], [6].

The genus *Zanclea* Gegenbaur, 1857, as well as others in the family Zancleidea, is known to be involved in epibiotic associations with different marine organisms [5]. *Zanclea timida* is associated with Octocorallia, and this symbiosis affects the morphology of both the hydroid and host [7]. Also, hydroids from the *Zanclea* genus inhabit the surface of scleractinian corals (Hexacorallia) as a substrate [8], [9]; *Zanclea gili*'s polyps were observed in association with scleractinian corals in Papua New Guinea, but the coral species have yet to be identified [4], and 2 new species associated with corals were recently described. *Zanclea margaritae* sp. nov. was found in specific association with the reef-building coral, *Acropora muricata*, on the Great Barrier Reef [10], [11], and *Zanclea sango* sp. nov., described from Okinawa, Japan, was associated with 3 other coral species, *Pavona divaricata*, *P. venosa*, and *Psammocora contigua* [6].

Although observations were made of the association between *Zanclea* sp. and scleractinian corals [4], [6], [8], [10], it is still unclear if the association is a species-specific symbiosis or a cosmopolitan association. In fact, to the present, there are very few studies related to the phylogeny of *Zanclea* species associated with corals, and there are no molecular analyses involving this species; hence, the phylogenetic position of the genus is still problematic. The genus *Zanclea* was studied with a classical morphological approach [12], and more recently with a molecular analysis [13], [14], using the 16S, 18S, and 28S molecular markers. Furthermore, no studies related to *Zanclea*-coral associations have been carried out in a wide area involving many coral species. This lack of knowledge leaves unresolved whether the *Zanclea*-scleractinia association is really a ‘novel’ species-specific symbiosis or there is a cosmopolitan *Zanclea* species complex associated with several coral species.

To understand the type of association between *Zanclea* and scleractinian corals, in the present study, we investigated the phylogeny of *Acropora*-associated *Zanclea*, and the *Zanclea* genus as a whole. Furthermore, we analyzed the presence and diversity of hydroids associated with different *Acropora* species from several locations in Taiwan, Australia, and Indonesia. Other genera of the Acroporidae were also included to understand how ubiquitous this association is. We hypothesized either that coral hosts present in different environments and locations would be associated with different types of hydroids or alternatively that hydroids would be cosmopolitan in their association.

## Materials and Methods

### Ethics statement

All necessary permits were obtained for the described field studies. Coral colonies were collected in Chinwan Inner Bay (CIB), Penghu, Taiwan, under permit number 90PBAF03751 assigned to the Penghu Marine Biology Research Center. The sampled coral colonies were collected non-destructively, and moved back to the wild after collecting the branches with hydroids from each colony. Samples in Kenting, Taiwan were collected as part of long-term ecological monitoring research through permits (nos. 1002901146 and 1010001032 for the years 2010 and 2011, respectively) assigned by the Kenting National Park Authority. Collection of samples in Australia was made possible by permission of the Great Barrier Reef Marine Park Authority for Orpheus Island, and in the case of Osprey Reef (Coral Sea), no permit was required at the time of collecting. Samples from Indonesia, Togian Islands were collected with permission of the Ministry of Forestry, Indonesia and Indonesian Institute of Sciences (LIPI).

### Sample collection

**Figure 1:**
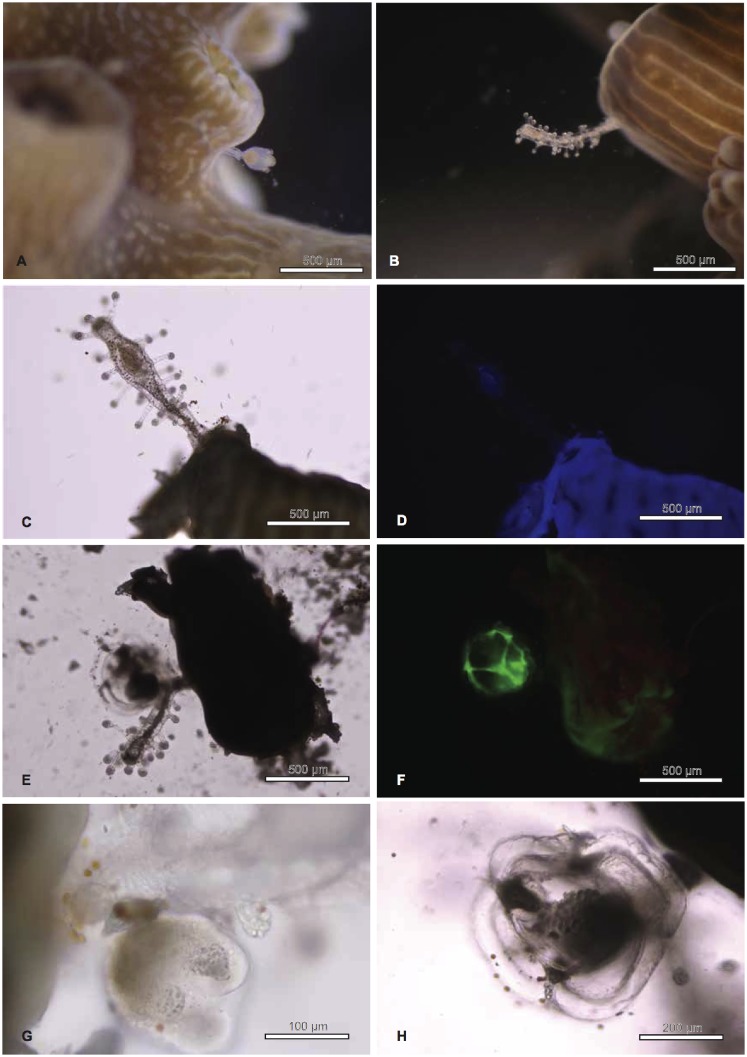
Micrographs showing different hydroids emerging from *Acropora muricata* axial corallites. A gonozooid showing an immature medusa (A) and gastrozooid (B). Micrographs of a gastrozooid attached to the axial corallites, showing ingested food, taken with normal light (C) and blue fluorescence (D). Blue fluorescence is present in the coral and in the food, but the hydroid itself shows none. Micrographs of a mature medusa and gastrozooid attached to the coral with normal (E) and combined green-red fluorescence (F). Unlike the gastrozooid, the mature medusa shows green fluorescence. Details of immature (G) and mature (H) medusae.

Coral colonies of *Acropora* spp. were surveyed *in situ* for the presence of hydroids in June 2011 at a subtropical coral community in Chinwan Inner Bay (CIB), Penghu, Taiwan. Four species of *Acropora* (*A. muricata*, *A. valida*, *A. humilis*, and *A. hyacinthus*) were selected for the hydroid survey and sampling, as these species are abundant in CIB. For each species, 15 to 32 colonies (Table 1) were checked *in situ* for the presence of hydroids underwater by disturbing the water near the colonies by hand, since hydroids present on coral branches do not retract when disturbed, unlike coral polyps. For other coral genera present at CIB besides *Acropora* (i.e., *Galaxea*, *Pavona*, *Goniastrea*, *Echinophyllia*, and *Montipora*), around 20∼30 colonies for each genus were also checked for the presence of hydroids, during 2 dives of 1 h each. After confirming the presence of hydroids in *Acropora* species, part of the colony with hydroids was collected and placed in an outdoor tank at the Penghu Marine Biology Research Center and Biodiversity Research Center, Academia Sinica (PMBRC-BRCAS) marine laboratory. For each species, hydroids from 3 or 4 colonies were sampled. Coral fragments (3∼4 cm, from the tip of each branch) full of hydroids from the colonies kept in the outdoor seawater tank were collected and immediately preserved in absolute ethanol for molecular analysis. Photographs of the hydroids attached to the coral skeleton (Fig. 1) were taken using microscopes (models CX31 and BX43, Olympus, Tokyo, Japan) with attached cameras (C5050 and DP72 CCD, Olympus, respectively) before fixing them in absolute ethanol for further analyses. A fragment of *Montipora* sp. was collected in Kenting (southern Taiwan) and fixed in ethanol after visually observing the associated hydroids.

**Table 1:** Information regarding coral samples surveyed *in situ* and collected from Chinwan Inner Bay (CIB), Penghu, Taiwan, and markers used for each sample.

Species	No. of colonies surveyed	No. of colonies with hydroids	No. of colonies sampled	Sample size	Sequences obtained		
					ITS	16S	28S
***Acropora muricata***	32	10	4	13	13	8	2
***Acropora humilis***	25	7	4	10	10	7	3
***Acropora valida***	15	3	3	9	9	5	3
***Acropora hyacinthus***	21	0	0	0	-	-	-
**Total**				**32**			

For each colony, 20 branches were collected.

In addition, several *Acropora* species (and some specimens of different genera of the Acroporidae) collected in Kenting, Taiwan; Tongian Island, Indonesia; and Osprey Reef and Orpheus Island, the Great Barrier Reef, Australia were also included in the molecular analysis.

### DNA extraction

Total genomic DNA from ethanol-fixed branches of 3 *Acropora* spp. collected at CIB, Penghu (Taiwan) and from a sample of *Montipora* sp. collected in Kenting (Taiwan) was extracted by the salting-out method [15]. From each ethanol-fixed branch, coral tissue together with the hydroids was scraped off using a scalpel. Scraped coral tissue was lysed overnight in a 2-ml Eppendorf tube with 200 µl lysis buffer (0.25 M Tris, 0.05 M EDTA at pH 8.0, 2% sodium dodecylsulfate, and 0.1 M NaCl) and 10 µl proteinase E (10 mg/ml) at 55°C in a water bath. NaCl (7 M, 210 µl) was added to the lysed tissue in the tube and mixed carefully by inverting the tube, and then the solution was transferred to a tube of a DNA spin column (Viogene-BioTek, Taipei, Taiwan) and centrifuged at 8000 rpm for 1 min. The lysate was washed twice with 500 µl of ethanol (70%) by centrifugation in each step at 8000 rpm for 1 min followed by an extra centrifugation step at 8000 rpm for 3 min to dry the spin column. The column was further dried at 37°C for 15 min, and in the final step, the DNA was eluted by adding 25 µl of TE buffer, with subsequent centrifugation twice at 13,000 rpm for 3 min.

### Primer designing, amplification, and sequencing

Target segments of portions of the mitochondrial 16S and nuclear 28S ribosomal genes, and the ITS region (partial 28S-ITS1-5.8S-ITS2-partial 18S) were amplified by a polymerase chain reaction (PCR). All primer sets were specifically designed to distinguish host DNA from symbiotic hydroid DNA. To design the primers, several sequences of hydroids (Hydrozoa, Hydroida, especially from the family Anthomedusae), different *Acropora* species, and some other scleractinian corals were downloaded from GenBank. All sequences were aligned using MEGA5 [16], and primers were designed using a 20∼25-bp fragment conserved within hydroids but not between hydroids and corals. Genomic DNA was amplified for the entire ITS region (670 bp) using the primer pair, hITSF (5′-GCC GAA AAG TTG ACC AAA CTT GAT C-3′) and hITSR (5′-AGC GGG TAG TCT TGT CTG ATC T-3′), and for ITS1 using the primer pair hITS1F (5′-TAC CGT TTG TCT CAT GAC AAA AAC C-3′) and hITS1R (5′-TAA AAG TTG TCA AGT GTT TAC TTT CA-3′). ITS1 primers were used to screen for the presence or absence of hydroids in the DNA, since these produced more-reliable amplification than the entire ITS primers. A ∼400-bp portion of 16S was amplified using the primer pair, h16SF (5′-TCA TTC GCC ATT TAA TTG ATG GAT A-3′) and h16SR (5′-TGT TTT CGA TAT GAT CTC TAR AAC AA-3′); and a highly variable ∼300-bp portion of 28S was amplified using the primer pair, h28SF (5′-AGG GAA GCG CAT GGA ATT AGC AAT G-3′) and h28SR (5′-AGC CCA AAA GAG CAT GTG CCG CGA C-3′).

All PCRs were set up in a volume of 50 µl: 5 µl PCR buffer (1×), 3 µl MgCl_2_ (1.5 mM), 1 µl of each dNTP (10 mM), 2 µl of each primer (2 µM), 2% DMSO, 0.2 µl of Taq enzyme mix, and approximately 5 µl of template DNA (20 ng/µl). The following PCR profiles were used: for the ITS and ITS1 regions, 1 cycle at 94°C for 2 min, 35 cycles at 94°C for 45 s, 55°C for 45 s, and 72°C for 90 s, and a final cycle at 72°C for 10 min; and for the 16S and 28S regions, 1 cycle at 94°C for 2 min, 35 cycles at 94°C for 30 s, 45°C for 1 min (for 28S, the temperature at this step was 47°C), and 72°C for 90 s, and a final cycle at 72°C for 10 min. All PCR products were electrophoresed in 1.5% agarose gels to assess the yield and directly sequenced. PCR products of the 28S region were purified before sequencing using a PCR purification kit (Qiagen) following the manufacturer's protocol. This step was necessary due to the presence of multiple DNA fragments, which were amplified with the primers.

All the sequences obtained from this study were submitted to DRYAD database with accession DOI: http://dx.doi.org/10.5061/dryad.g0b20.

### Phylogenetic analyses

Identification of hydroid phylogenetic positions at the genus and species levels was carried out using the 2 genetic markers: mitochondrial 16S and nuclear 28S. Diversity within the hydroid sequences was analyzed using the 16S, 28S, and ITS markers.

**Figure 2:**
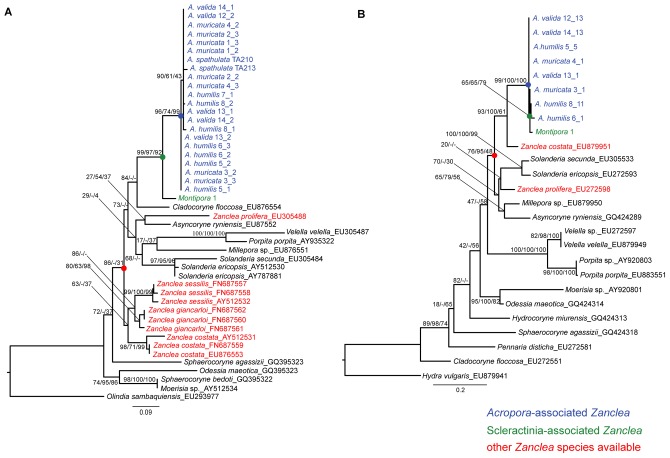
Phylogenetic tree based on 16S (A) and 28S (B) gene sequences. The topology was inferred using a maximum-likelihood (ML) analysis. Numbers on main branches show percentages of the SH-like value for the ML analysis and bootstrap support with 1000 repetitions for a Bayesian analysis and Neighbor-joining analysis. *Acropora*-associated *Zanclea* samples are highlighted in blue, other Scleractinia-associated hydroids are in green, and other available *Zanclea* species are in red. Nodes supporting the clade including *Acropora*-associated hydroids, all Scleractinia-associated hydroids, and all *Zanclea* species are respectively highlighted in blue, green, and red.

Reference sequences obtained through a search using BLAST (http://www.ncbi.nlm.nih.gov/blast/Blast.cgi) were downloaded from GenBank, and species were also chosen based on recent molecular taxonomic work [13], [14], [17] for comparison and alignment (Table S1). The initial sequence assembly and analysis were conducted using SeqMan (DNASTAR; http://www.dnastar.com). Sequences were aligned using MEGA5 [16] and were adjusted visually. Phylogenetic relationships based on 16S and 28S were inferred using Neighbor-joining (NJ), maximum-likelihood (ML), and Bayesian analyses (BA). The NJ analysis was performed with the Kimura 2-parameter (K2P) model of nucleotide substitutions [18], and bootstrap analyses with 1000 replicates were conducted in order to assess the node support. For the ML and Bayesian analyses, the optimal molecular evolution model was determined using the Akaike information criterion (AIC) [19], performed using MODELTEST 2.3 [20] and PAUP 4.0b10 [21]. The most suitable models selected by the AIC for the analysis were GTR+G (gamma = 0.5280 and p-invar = 0) for 28S and AKY+I+G (gamma = 0.4607 and p-invar = 0.2783) for 16S. The ML analysis was performed in PhyML 3.0 [22], using the Shimodaira and Hasegawa (SH)-like test to check the support of each inner branch. The Bayesian analysis was performed in MrBayes [23], running a Markov chain for a minimum 10^6^ and a maximum of 2×10^6^ generations, with a tree saved every 100 generations, and the first 25% of generations discarded as burn-in. The final alignment of 16S (382 bp) used 22 hydroid sequences available in GenBank and 23 sequences of hydroids obtained in this study from *A. muricata*, *A. humilis*, *A. valida*, *A. spathulata* and *Montipora* sp. (see Fig. 2 and Table S1 for details). For the final alignment of 28S (273 bp), 16 hydroid sequences available in GenBank and 9 sequences of hydroid obtained in this study from *A. muricata*, *A. humilis*, *A. valida*, and *Montipora* sp. were used (see Fig. 2 and Table S1 for details). Sequences corresponding to *Olindias sambaquiensis* and *Hydra vulgaris* were chosen as outgroups. For the final ITS alignment, 32 sequences of hydroids associated with *A. muricata*, *A. humilis*, and *A. valida* collected at CIB, Penghu, Taiwan were used.

Different matrices of p-distances based on 16S and 28S, within specimens analyzed in the present work (i.e., Scleractinia-associated hydroids), and between and within other hydroid species, corresponding to *Zanclea* sp. and *Solanderia* sp. sequences available in GenBank, were generated with MEGA5 [16]. For 16S and 28S analyses, 8 hydroids sequenced from *A. muricata*, 7 from *A. humilis*, 5 from *A. valida*, 2 from *A. spathulata*, and 1 from *Montipora* sp. and 9 hydroid sequences from *A. muricata*, *A. humilis*, *A. valida*, and *Montipora* sp. were respectively used. In case of 28S, only 1 sequence for each species was available in GenBank. Analyses were conducted using the K2P model [18].

To analyze the hydroid presence in different *Acropora* species (and in other members of the Acroporidae) from different locations, the ITS1 fragment was chosen as a marker for screening. The entire sample size was amplified using an ITS1 primer pair specific for hydroids to screen for the presence or absence of a band specific to hydroid DNA in the total DNA samples on 2% agarose gels (Table 2). PCR products of only some samples with positive amplification for the presence of hydroids were sequenced. To ensure that the bands seen in the gel effectively corresponded to hydroid DNA, and not to the coral host, some samples were randomly selected for sequencing. All sequences obtained were submitted to an NCBI BLAST search to see if they matched Hydrozoa and/or Scleractinia sequences.

**Table 2:** Information regarding different *Acropora* species (and other members of the Acroporidae) collected from different locations, and analyzed for the presence of hydroids.

Species	Location	Sample size (samples amplified using ITS1)	Sample with hydroids (presence in ITS1 gel)	Sequences obtained		
				ITS1	16S	28S
***Acropora muricata***	Osprey Reef (Australia) (1 sample), Penghu Islands (Taiwan) (13)	14	14	14	8	2
***Acropora humilis***	Osprey Reef (Australia) (8 samples), Penghu Islands (10) and Kenting (Taiwan) (2)	20	20	13	7	3
***Acropora valida***	Penghu Islands (9 samples) and Kenting (Taiwan) (2)	11	10	9	5	3
**Acropora hycinthus**	Kenting (Taiwan)	1	1	1	-	-
***Acropora spathulata***	Orpheus Island and Osprey Reef (Australia)	7	4	1	2	-
***Acropora millepora***	Orpheus Island (Australia)	2	2	1	-	-
***Acropora aspera***	Orpheus Island (Australia)	2	1	1	-	-
***Acropora pulchra***	Orpheus Island (Australia), Kenting (Taiwan)	2	1	1	-	-
***Acropora samoensis***	Osprey Reef (Australia)	4	3	1	-	-
***Acropora granulosa***	Osprey Reef (Australia)	4	2	1	-	-
***Acropora loripes***	Osprey Reef (Australia)	8	6	1	-	-
***Acropora speciosa***	Osprey Reef (Australia)	2	1		-	-
***Acropora gemmifera***	Osprey Reef (Australia), Kenting (Taiwan)	11	7	1	-	-
***Acropora divaricata***	Kenting (Taiwan)	2	2	-	-	-
***Acropora sp.***	Orpheus Island and Osprey Reef (Australia), Kenting (Taiwan)	14	8	-	-	-
***Astreopora spp.***	Lyudao and Kenting (Taiwan)	6	3	-	-	-
***Anacropora spp.***	Kenting (Taiwan), Tongian Island (Indonesia)	5	4	-	-	-
***Montipora spp.***	Kenting (Taiwan)	9	6	3	1	1
***Isopora palifera***	Kenting (Taiwan)	18	3	-	-	-
		**Total: 142**				

The entire sample size was amplified using ITS1, and screened for the occurrence of hydroids, detected using the presence of a specific band in the gel. Among samples with a positive result for the presence of the band, ITS1, 16S, and 28S sequences of some samples were also obtained. Numbers of samples with the presence of hydroids and the relative sequences obtained are presented. In *Acropora* samples collected at Penghu (Taiwan) and in one *Montipora* sample collected at Kenting (Taiwan), the presence of hydroids was visually detected. For these samples, we obtained the sequence of the entire ITS fragment.

## Results

### Inter-colony variations in hydroid associations

Results from the visual survey carried out at CIB, Penghu, Taiwan showed the presence of hydroids in several colonies of *A. muricata*, *A. humilis*, and *A. valida* (see Fig. 1 for example). The abundance of associated hydroids varied both within and between species. While most of the colonies had high densities (>30 hydroids per branch), the lowest density was <5 hydroids per branch. No particular pattern was observed in the distribution of hydroids on the colonies or in the distribution of colonies with and without symbiotic hydroids. We found 10, 7, and 3 colonies with hydroid in a total of 32, 25, and 15 colonies surveyed for *A. muricata*, *A. humilis*, and *A. valida*, respectively (Table 1). We found no hydroids associated with the coral *A. hyacinthus* during the underwater *in situ* survey, hence this species was not collected or included in the analysis (Table 1). Furthermore, we found no hydroids on other genera visually examined at CIB (i.e., *Galaxea*, *Pavona*, *Goniastrea*, *Echinophyllia*, and *Montipora*).

### Phylogenetic analyses

In total, 142 coral samples, including 104 from the genus *Acropora* were used for the present analysis (Table 2). *Acropora* specimens surveyed and collected at CIB, Penghu Islands (Taiwan) are listed in Table 1.

At first, hydroid sequences obtained in the present analysis from different scleractinian corals were identified using NCBI BLAST. Sequences of 16S and 28S were closely related to sequences corresponding to the genera *Zanclea* (with high similarities of >99%) and *Solanderia*. The ITS BLAST search found that sequences of hydroids from this study had high similarity to the genus *Millepora*, since ITS sequences of other genera are not yet available in GenBank.

According to analyses based on the 16S and 28S ribosomal genes (Fig. 2A, B), sequences of different *Zanclea* species, including sequences of hydroids from this study, did not cluster together. In the 16S analysis, sequences form *Z. costata*, the type species of the genus, formed a monophyletic clade with *Z. sessilis* and *Z. giancarloi*. However, the only sequence from *Z. prolifera* clustered with the species *Asyncoryne ryniensis*. For the 28S analysis, only 2 sequences of *Zanclea* were available in GenBank, and they did not cluster together. In this case, the type species, *Z. costata*, formed a monophyletic group with the hydroid from this study. Overall results showed non-monophyly of the genus *Zanclea*. However, hydroid sequences from this work were found closely related to other available *Zanclea* species (Fig. 2A, B, in red).

The phylogenetic analysis determined a monophyletic status for *Acropora*-associated *Zanclea* with both the 16S and 28S genes (Fig. 2A, B, in blue), with high support for the node. The phylogenetic topology suggested that the 4 *Acropora* species (*A. muricata*, *A. valida*, *A. humilis*, and *A. spathulata*) were associated with the same hydroid species. All Scleractinia-associated hydroids were also monophyletic (Fig. 2A, B, in green).

Alignment of the ITS marker showed remarkable uniformity and confirmed the results obtained with the 16S and 28S rDNA markers. The final ITS alignment consisted of 670 bp with no indels and only 1 single-nucleotide polymorphism (SNP).

Pairwise distance estimates of evolutionary divergence based on 16S and 28S were calculated between hydroid sequences from this study (Scleractinia-associated *Zanclea*) with 6 other closely related hydroid species. Results (Table 3, 4) supported the close relationship of hydroids surveyed in the present analysis. Intra-group distances (with a mean pairwise distance of 0.009 for 16S and 0.006 for 28S) within Scleractinia-associated *Zanclea* did not overlap with interspecific distances between sequences of this study and sequences downloaded from the NCBI database (Table 3, 4).

**Table 3:** Intra- and interspecific estimates of evolutionary divergence between Scleractinia-associated *Zanclea* and other species, expressed as the pairwise distance based on 16S markers.

P-distance	Scleractinia-associated hydroid	*Z. sessilis*	*Z. costata*	*Z. giancarloi*	*Z. prolifera*	*Solanderia secunda*	*S. ericopsis*
**Scleractinia-associated hydroid**	**0.009 (±0.002)**						
***Z. sessilis***	0.114 (±0.017)	**0.013 (±0.005)**					
***Z. costata***	0.135 (±0.019)	0.085 (±0.015)	**0.029 (±0.007)**				
***Z. giancarloi***	0.101 (±0.015)	0.055 (±0.011)	0.072 (±0.013)	**0.013 (±0.005)**			
***Z. prolifera***	0.173 (±0.023)	0.149 (±0.021)	0.147 (±0.021)	0.132 (±0.020)	**0.00(** * **)**		
***Solanderia secunda***	0.146 (±0.021)	0.143 (±0.021)	0.137 (±0.019)	0.141 (±0.021)	0.184 (±0.023)	**0.00(** * **)**	
***S. ericopsis***	0.128 (±0.018)	0.106 (±0.017)	0.108 (±0.017)	0.104 (±0.017)	0.171 (±0.023)	0.073 (±0.014)	**0.008 (±0.005)**

Intragroup distances (in bold) of Scleractinia-associated *Zanclea* do not overlap with interspecific distances.

* Only 1 sequence was available for analysis.

Numbers of base substitutions per site from averaging all sequence pairs between and within groups are shown. Standard error estimates are shown in brackets.

**Table 4:** Intra- and interspecific estimates of evolutionary divergence between Scleractinia-associated *Zanclea* and other species, expressed as the pairwise distance based on 28S markers.

P-distance	Scleractinia-associated hydroid	*Z. costata*	*Z. prolifera*	*S. secunda*	*S. ericopsis*
**Scleractinia-associated hydroid**	**0.006 (±0.002)**				
***Z. costata***	0.110 (±0.023)	**0.00(** * **)**			
***Z. prolifera***	0.179 (±0.030)	0.154 (±0.027)	**0.00(** * **)**		
***S. secunda***	0.158 (±0.029)	0.148 (±0.027)	0.143 (±0.027)	**0.00(** * **)**	
***S. ericopsis***	0.165 (±0.031)	0.144 (±0.027)	0.144 (±0.026)	0.058 (±0.016)	**0.00(** * **)**

Intragroup distances (in bold) of Scleractinia-associated *Zanclea* do not overlap with interspecific distances.

* Only 1 sequence was available for analysis.

Numbers of base substitutions per site from averaging all sequence pairs between and within groups are shown. Standard error estimates are shown in brackets.

Specific primer pairs that amplified ITS1, used to screen for the occurrence of symbiotic hydroids through the presence-absence of a specific band in the agarose gel, confirmed their presence in all *Acropora* species and in all locations analyzed (Table 2). We also detected the presence of hydroids in other Acroporidae genera (*Astreopora*, *Anacropora*, *Montipora*, and *Isopora*) (Table 2; all species analyzed for the presence of hydroids are listed in Table S2). Among samples selected for sequencing, no sequences corresponded to the coral hosts. Sequences of ITS1 obtained from a larger dataset also confirmed the low diversity found using ITS, 16S, and 28S.

## Discussion

Results from this study showed that hydroids associated with different scleractinian corals are cosmopolitan, and the *Zanclea* genus is not monophyletic. Instead, species of *Zanclea* associated with several *Acropora* species were found to be monophyletic with very low genetic diversity. Evidence suggests that hydroids in all *Acropora* samples analyzed belonged to the genus *Zanclea*, and this was a genus-specific association. We detected the presence of hydroids using a specific primer pair for ITS1, associated with different *Acropora* corals and some other members of the Acroporidae in a large geographical area across different latitudes: Taiwan, Indonesia, and the Great Barrier Reef. The presence of the medusa life stage and continuous currents between the Great Barrier Reef and Taiwan may enhance the large-scale dispersal of this species. Moreover, this species found a perfect substrate on *Acropora* corals, which are widespread, making it possible for *Zanclea* to colonize a wide area.

A recent study showed the presence of the hydroid, *Zanclea margaritae*, in scleractinian corals, associated with only 1 *Acropora* species, *A. muricata*, initially at Heron Island, and then later at Orpheus Island of the Great Barrier Reef [10]. Furthermore, another *Zanclea* species, *Z. sango*, was found to be associated with *Pavona divaricata*, *P. venosa*, and *Psammocora contigua* in Okinawa, Japan [6]. The main feature that distinguishes the 2 species is the lack of a perisarc (a chitinous exoskeleton covering the hydrorhiza) in *Z. margaritae*, which is considered to be due to advanced integration with the host and a stricter symbiosis [5]. The above studies used only classical taxonomy to identify the species with no molecular analyses.

Initial surveys carried out at CIB, Penghu, Taiwan, for hydroids on 4 *Acropora* species (*A. muricata, A. humilis, A. valida*, and *A. hyacinthus*) revealed their presence on 3 species (*A. muricata, A. humilis*, and *A. valida*). Other coral species present at the same location were also checked, but no hydroids were detected.

Although we can confirm the genus identity of the hydroids found in this study (i.e. *Zanclea*), and we suspect that the species may be *Z. margaritae* since this species was found in association with *A. muricata* in previous studies, morphological analysis are needed to confirm the exact hydroid species.

According to molecular analyses of the 16S and 28S ribosomal genes (Fig. 2A, B), the genus *Zanclea* is not monophyletic. However, sequences obtained from this study were found to be very closely related to other *Zanclea* species available in GenBank, as they were included in the smallest monophyletic group encompassing all *Zanclea* species used for the analysis (Fig. 2A, B, in red), and this group showed high support for both topologies. However, according to the 16S analysis (Fig. 2A), *Z. costata*, the type species of the genus, formed a monophyletic clade with *Z. giancarloi* and *Z. sessilis*, and this seems to be the “true” *Zanclea* clade. The other *Zanclea* sp. present in the topology, *Z. prolifera*, was proposed to actually belong to the genus *Asyncoryne* [14], and this is consistent with our analysis. These latter results suggest the possibility that the species in the present study does not belong to the genus *Zanclea*, but to an as yet undescribed genus, that needs to be better studied. On the other hand, in the 28S analysis (Fig. 2B), sequences from this study clustered with *Z. costata*, although only 1 sequence was available for this marker. Although only around 300 bp of sequences downloaded form GenBank was used for the 28S phylogenetic analysis, the observed pattern was consistent with a previous analysis involving the complete 28S gene [14]. Previous studies [13], [14], [17] suggested similar patterns for both the 16S and 28S analyses.

In the present work, we still consider the species from this study to be *Zanclea*, since all described hydroids associated with Scleractinia are considered to be *Zanclea* according to morphologic analyses [6], [10], [11], but we are aware that the *Zanclea* question needs to be further addressed with both molecular and morphologic tools.

The molecular phylogeny obtained using 16S and 28S ribosomal DNA showed the monophyletic status of *Zanclea* associated with *Acropora* corals (Fig. 2A, B, in blue), suggesting very low diversity between them. Hydroids associated with scleractinians were also monophyletic (Fig. 2A, B, in green); however, due to the small sample size of non-*Acropora*-associated *Zanclea* used for the phylogenetic analysis, this latter result needs to be confirmed with further analyses.

The low diversity of Scleractinia-associated *Zanclea* was confirmed by the pairwise distance analysis based on the 16S and 28S markers (Table 3, 4). Within-species values (Table 3, 4 in bold) for Scleractinia-associated hydroids (0.009 and 0.006 base substitutions per site for 16S and 28S, respectively) were always lower than between-species values. Intra-species diversity in other *Zanclea* spp. analyzed was higher, and this is consistent with the conclusion of only 1 species in the samples analyzed. The analysis based on the ITS showed no diversity between hydroids associated with *A. muricata*, *A. humilis*, and *A. valida* collected at CIB, Penghu, Taiwan. Furthermore, analysis of the ITS1 fragment of the total ITS also confirmed the low diversity between hydroids in coral samples analyzed from a larger dataset including different locations in Australia, Indonesia, and Taiwan. Results clearly suggest the presence of a single genetic group forming this novel symbiotic relationship with at least 14 *Acropora* species over a large geographic area.

Results of the ITS analysis might have been due to the low resolution of this marker. Although the ITS might not be a good marker to resolve species boundaries, the 16S and 28S markers were previously used to distinguish different hydroid species, including some *Zanclea* species [13], [14]. Furthermore, our phylogenetic analysis involving 16S (Fig. 2A) confirmed the latter as a marker able to distinguish other *Zanclea* species available in GenBank. However, since the present work is the first molecular study involving *Acropora*-associated hydroids, there is a need to develop a number of different markers to distinguish putative intra-groups.

### Conclusions

Although associations between hydroids and Scleractinia are known, there is a lack of molecular data in previous studies. The present work is an important first attempt at characterizing this type of symbiosis at the molecular level. In fact, we suggest that this symbiosis is more generalist than described in a previous study [10], involving different host genera. Further analyses are necessary, especially involving other scleractinian genera, to understand how evolutionary trends in the *Zanclea* genus are related to stricter symbioses, relationships with coral taxa, and geographic distribution patterns.

## Supporting Information

Table S1 Information on GenBank accession numbers of hydroid sequences used for the phylogenetic analysis.
(DOC)

Table S2 Sample list of genomic DNA of coral samples used from the database of the CREEG laboratory to search for the presence or absence of hydroids (code, species, location name). “x” denotes the presence of hydroids. For some samples, the sample was sequenced to confirm the presence of hydroids.
(DOC)
