# Supplementary material for: Molecular Evidence Shows Low Species Diversity of Coral-Associated Hydroids in Acropora Corals
Source: PLoS One. 2012 Nov 29;7(11):e50130. doi: 10.1371/journal.pone.0050130 (PMC3510231; doi:10.1371/journal.pone.0050130)
Supplement: Table S1 — Information on GenBank accession numbers of hydroid sequences used for the phylogenetic analysis. (DOC) [file pone.0050130.s001.doc]

**Table S1. Information on GenBank accession numbers of hydroid sequences used for the phylogenetic analysis.**

| **Species** | **Location** | **Accession no.** |
| --- | --- | --- |
| **Sequences corresponding to 16S:** | | |
| ***Asyncoryne ryniensis*** | Japan: Seto Marine Biological Station, CnidTOL | EU876552 |
| ***Cladocoryne floccosa*** | Japan: Seto Marine Biological Station, CnidTOL | EU876554 |
| ***Millepora sp.*** |  | EU876551 |
| ***Moerisia sp.*** | USA: San Francisco Bay Region, CA | AY512534 |
| ***Odessia maeotica*** | France: Portiragnes | GQ395324 |
| ***Olindias sambaquiensis*** |  | EU293977 |
| ***Porpita porpita*** |  | AY935322 |
| ***Solanderia ericopsis*** | New Zealand: Leigh | AY512530 |
| ***Solanderia ericopsis*** | New Zealand: Leigh | AY787881 |
| ***Solanderia secunda*** |  | EU305484 |
| ***Sphaerocoryne agassizii*** | USA: Florida | GQ395323 |
| ***Sphaerocoryne bedoti*** | Panama | GQ395322 |
| ***Velella velella*** | France: Villefranche-sur-Mer | EU305487 |
| ***Zanclea costata*** | France, Marie-de-la-Mer, Mediterranean Sea | AY512531 |
| ***Zanclea costata*** | France: Marie-de-la-Mer, Mediterranean | EU876553 |
| ***Zanclea costata*** | France: beach near Sainte-Marie-la-Mer | FN687559 |
| ***Zanclea giancarloi*** | France: Banyuls-sur-Mer | FN687560 |
| ***Zanclea giancarloi*** | France: La Ciotat | FN687561 |
| ***Zanclea giancarloi*** | Spain: Guipuzcoa, Fuenterrabia | FN687562 |
| ***Zanclea prolifera*** |  | EU305488 |
| ***Zanclea sessilis*** | Spain: Mallorca, Cala Murada | AY512532 |
| ***Zanclea sessilis*** | France: Roscoff | FN687557 |
| ***Zanclea sessilis*** | Spain: San Sebastian | FN687558 |
| **Sequences corresponding to 28S:** | | |
| ***Asyncoryne ryniensis*** | Japan | GQ424289 |
| ***Cladocoryne floccosa*** | Brazil: Ilhabela, Praia do Sino | EU272551 |
| ***Hydra vulgaris*** | Argentina: Arroyo San Claudio, Partido de Coronel Pringles | EU879941 |
| ***Hydrocoryne miurensis*** | Japan | GQ424313 |
| ***Millepora* sp.** |  | EU879950 |
| ***Moerisia* sp.** | USA: San Francisco Bay Region, CA | AY920801 |
| ***Odessia maeotica*** | France: Portiragnes | GQ424314 |
| ***Pennaria disticha*** | Spain: Mallorca, Cala Murada | EU272581 |
| ***Porpita porpita*** |  | EU883551 |
| ***Porpita* sp.** |  | AY920803 |
| ***Solanderia ericopsis*** |  | EU272593 |
| ***Solanderia secunda*** |  | EU305533 |
| ***Sphaerocoryne agassizii*** | USA: Florida | GQ424318 |
| ***Zanclea costata*** | France: Marie-de-la-Mer, Mediterranean | EU879951 |
| ***Zanclea prolifera*** |  | EU272598 |
| **Sequences corresponding to ITS:** | | |
| ***Craspedacusta sowerbyi*** | Germany: Loebejuen | FJ423633 |
| ***Distichopora sp.*** |  | U65483 |
| **Eukaryota; environmental samples** | South China Sea | GU941346 |
| **Eukaryota; environmental samples** | South China Sea | GU941910 |
| **Eukaryota; environmental samples** | South China Sea | GU942276 |
| **Eukaryota; environmental samples** | South China Sea | GU942282 |
| **Eukaryota; environmental samples** | South China Sea | GU942419 |
| ***Hydra vulgaris*** |  | GU722712 |
| ***Millepora exaesa*** |  | U65484 |
| ***Millepora sp.*** | Israel: Elat | AJ515059 |
| ***Millepora sp.*** | Israel: Elat | AJ515060 |
