# Supplementary material for: Molecular Evidence Shows Low Species Diversity of Coral-Associated Hydroids in Acropora Corals
Source: PLoS One. 2012 Nov 29;7(11):e50130. doi: 10.1371/journal.pone.0050130 (PMC3510231; doi:10.1371/journal.pone.0050130)
Supplement: Table S2 — Sample list of genomic DNA of coral samples used from the database of the CREEG laboratory to search for the presence or absence of hydroids (code, species, location name). “x” denotes the presence of hydroids. For some samples, the sample was sequenced to confirm the presence of hydroids. (DOC) [file pone.0050130.s002.doc]

**Table S2. Sample list of genomic DNA of coral samples used from the database of the CREEG laboratory to search for the presence or absence of hydroids (code, species, location name).** “x” denotes the presence of hydroids. For some samples, the sample was sequenced to confirm the presence of hydroids.

| **Code** | **Species** | **Location** | **Presence of hydroids**  **(ITS marker)** | **Sequence** | | |
| --- | --- | --- | --- | --- | --- | --- |
|  |  |  |  | ITS1 | 16S | 28S |
| **TA210** | *Acropora spathulata* | Orpheus Island, Australia | x |  | x |  |
| **TA211** | *Acropora millepora* | Orpheus Island, Australia | x | x |  |  |
| **TA213** | *Acropora spathulata* | Orpheus Island, Australia | x | x | x |  |
| **TA218** | *Acropora millepora* | Orpheus Island, Australia | x |  |  |  |
| **TA224** | *Acropora aspera* | Orpheus Island, Australia | x | x |  |  |
| **TA225** | *Acropora pulchra* | Orpheus Island, Australia | x |  |  |  |
| **TA247** | *Acropora aspera* | Orpheus Island, Australia |  |  |  |  |
| **TA274** | *Acropora* sp. | Orpheus Island, Australia |  |  |  |  |
| **TA1** | *Acropora samoensis* | Osprey Reef, Australia | x | x |  |  |
| **TA4** | *Acropora granulosa* | Osprey Reef, Australia |  |  |  |  |
| **TA6** | *Acropora loripes* | Osprey Reef, Australia | x |  |  |  |
| **TA7** | *Acropora loripes* | Osprey Reef, Australia | x | x |  |  |
| **TA8** | *Acropora granulosa* | Osprey Reef, Australia | x |  |  |  |
| **TA10** | *Acropora loripes* | Osprey Reef, Australia | x |  |  |  |
| **TA11** | *Acropora loripes* | Osprey Reef, Australia | x |  |  |  |
| **TA12** | *Acropora samoensis* | Osprey Reef, Australia | x |  |  |  |
| **TA13** | *Acropora speciosa* | Osprey Reef, Australia | x |  |  |  |
| **TA14** | *Acropora granulosa* | Osprey Reef, Australia | x | x |  |  |
| **TA15** | *Acropora speciosa* | Osprey Reef, Australia |  |  |  |  |
| **TA16** | *Acropora loripes* | Osprey Reef, Australia |  |  |  |  |
| **TA17** | *Acropora spathulata* | Osprey Reef, Australia |  |  |  |  |
| **TA19** | *Acropora spathulata* | Osprey Reef, Australia | x |  |  |  |
| **TA20** | *Acropora humilis* | Osprey Reef, Australia | x |  |  |  |
| **TA21** | *Acropora humilis* | Osprey Reef, Australia | x |  |  |  |
| **TA22** | *Acropora gemmifera* | Osprey Reef, Australia | x |  |  |  |
| **TA26** | *Acropora loripes* | Osprey Reef, Australia | x |  |  |  |
| **TA27** | *Acropora loripes* | Osprey Reef, Australia | x |  |  |  |
| **TA34** | *Acropora loripes* | Osprey Reef, Australia |  |  |  |  |
| **TA35** | *Acropora spathulata* | Osprey Reef, Australia | x |  |  |  |
| **TA36** | *Acropora spathulata* | Osprey Reef, Australia |  |  |  |  |
| **TA38** | *Acropora humilis* | Osprey Reef, Australia | x | x |  |  |
| **TA39** | *Acropora spathulata* | Osprey Reef, Australia |  |  |  |  |
| **TA42** | *Acropora humilis* | Osprey Reef, Australia | x |  |  |  |
| **TA43** | *Acropora humilis* | Osprey Reef, Australia |  |  |  |  |
| **TA49** | *Acropora samoensis* | Osprey Reef, Australia | x |  |  |  |
| **TA50** | *Acropora granulosa* | Osprey Reef, Australia |  |  |  |  |
| **TA89** | *Acropora humilis* | Osprey Reef, Australia |  |  |  |  |
| **TA92** | *Acropora humilis* | Osprey Reef, Australia | x |  |  |  |
| **TA93** | *Acropora humilis* | Osprey Reef, Australia | x | x |  |  |
| **TA116** | *Acropora sp.* | Osprey Reef, Australia | x |  |  |  |
| **TA133** | *Acropora sp.* | Osprey Reef, Australia |  |  |  |  |
| **TA138** | *Acropora muricata* | Osprey Reef, Australia | x | x |  |  |
| **TA146** | *Acropora sp* | Osprey Reef, Australia | x |  |  |  |
| **TA148** | *Acropora sp* | Osprey Reef, Australia |  |  |  |  |
| **TA149** | *Acropora sp* | Osprey Reef, Australia |  |  |  |  |
| **TA163** | *Acropora gemmifera* | Osprey Reef, Australia | x |  |  |  |
| **TA168** | *Acropora gemmifera* | Osprey Reef, Australia |  |  |  |  |
| **TA186** | *Acropora gemmifera* | Osprey Reef, Australia |  |  |  |  |
| **TA187** | *Acropora gemmifera* | Osprey Reef, Australia | x |  |  |  |
| **TA188** | *Acropora gemmifera* | Osprey Reef, Australia | x |  |  |  |
| **TA189** | *Acropora gemmifera* | Osprey Reef, Australia |  |  |  |  |
| **TA190** | *Acropora sp.* | Osprey Reef, Australia | x |  |  |  |
| **TA191** | *Acropora samoensis* | Osprey Reef, Australia |  |  |  |  |
| **TA193** | *Acropora gemmifera* | Osprey Reef, Australia |  |  |  |  |
| **TA195** | *Acropora gemmifera* | Osprey Reef, Australia | x |  |  |  |
| **Acr3** | *Acropora* sp. | Kenting, Taiwan | x | x |  |  |
| **Acr1109** | *Acropora hycinthus* | Kenting, Taiwan | x |  |  |  |
| **Acr1110** | *Acropora humilis* | Kenting, Taiwan |  |  |  |  |
| **Acr1246** | *Acropora* sp*.* | Kenting, Taiwan | x |  |  |  |
| **Acr1248** | *Acropora* sp. | Kenting, Taiwan |  |  |  |  |
| **Acr1249** | *Acropora* sp. | Kenting, Taiwan | x |  |  |  |
| **Acr1250** | *Acropora* sp. | Kenting, Taiwan |  |  |  |  |
| **Acr1251** | *Acropora* sp. | Kenting, Taiwan | x |  |  |  |
| **Acr1252** | *Acropora* sp. | Kenting, Taiwan | x |  |  |  |
| **Acr1255** | *Acropora divaricata* | Kenting, Taiwan | x |  |  |  |
| **Acr1257** | *Acropora pulchra* | Kenting, Taiwan |  |  |  |  |
| **Acr1258** | *Acropora valida* | Kenting, Taiwan |  |  |  |  |
| **Acr1260** | *Acropora divaricata* | Kenting, Taiwan | x |  |  |  |
| **Acr1343** | *Acropora humilis* | Kenting, Taiwan | x | x |  |  |
| **Acr1344** | *Acropora gemmifera* | Kenting, Taiwan | x |  |  |  |
| **Acr1345** | *Acropora valida* | Kenting, Taiwan | x |  |  |  |
| **Acr1346** | *Acropora gemmifera* | Kenting, Taiwan | x | x |  |  |
| **Ast2060** | *Astreopora explanata* | Lyudao, Taiwan | x |  |  |  |
| **Ast3524** | *Astreopora* sp. | Taiwan |  |  |  |  |
| **Ast4076** | *Astreopora explanata* | Kenting, Taiwan |  |  |  |  |
| **Ast4077** | *Astreopora explanata* | Kenting, Taiwan |  |  |  |  |
| **Ast4078** | *Astreopora myriophthalma* | Kenting, Taiwan | x |  |  |  |
| **Ast4079** | *Astreopora myriophthalma* | Kenting, Taiwan | x |  |  |  |
| **Ana3368** | *Anacropora forbesi* | Kenting, Taiwan | x |  |  |  |
| **Ana4072** | *Anacropora* sp. | Tongian Island, Indonesia | x |  |  |  |
| **Ana4073** | *Anacropora matthai* | Tongian Island, Indonesia | x |  |  |  |
| **Ana4074** | *Anacropora* sp. | Tongian Island, Indonesia | x |  |  |  |
| **Ana4075** | *Anacropora* sp. | Tongian Island, Indonesia |  |  |  |  |
| **Mon1** | *Montipora* sp. | Kenting, Taiwan | x | x | x | x |
| **Mon4525** | *Montipora foliosa* | Kenting, Taiwan | x |  |  |  |
| **Mon4528** | *Montipora foliosa* | Kenting, Taiwan |  |  |  |  |
| **Mon4539** | *Montipora faveolata* | Kenting, Taiwan | x | x |  |  |
| **Mon4552** | *Montipora foliosa* | Kenting, Taiwan | x | x |  |  |
| **Mon4553** | *Montipora mollis* | Kenting, Taiwan | x |  |  |  |
| **Mon4554** | *Montipora aeguituberculata* | Kenting, Taiwan |  |  |  |  |
| **Mon4563** | *Montipora aeguituberculata* | Kenting, Taiwan | x |  |  |  |
| **Mon4567** | *Montipora foliosa* | Kenting, Taiwan |  |  |  |  |
| **Iso716** | *Isopora palifera* | Kenting, Taiwan |  |  |  |  |
| **Iso717** | *Isopora palifera* | Kenting, Taiwan |  |  |  |  |
| **Iso718** | *Isopora palifera* | Kenting, Taiwan |  |  |  |  |
| **Iso721** | *Isopora palifera* | Kenting, Taiwan |  |  |  |  |
| **Iso723** | *Isopora palifera* | Kenting, Taiwan | x |  |  |  |
| **Iso724** | *Isopora palifera* | Kenting, Taiwan |  |  |  |  |
| **Iso725** | *Isopora palifera* | Kenting, Taiwan | x |  |  |  |
| **Iso726** | *Isopora palifera* | Kenting, Taiwan |  |  |  |  |
| **Iso727** | *Isopora palifera* | Kenting, Taiwan | x |  |  |  |
| **Iso728** | *Isopora palifera* | Kenting, Taiwan |  |  |  |  |
| **Iso731** | *Isopora palifera* | Kenting, Taiwan |  |  |  |  |
| **Iso732** | *Isopora palifera* | Kenting, Taiwan |  |  |  |  |
| **Iso733** | *Isopora palifera* | Kenting, Taiwan |  |  |  |  |
| **Iso735** | *Isopora palifera* | Kenting, Taiwan |  |  |  |  |
| **Iso736** | *Isopora palifera* | Kenting, Taiwan |  |  |  |  |
| **Iso737** | *Isopora palifera* | Kenting, Taiwan |  |  |  |  |
| **Iso738** | *Isopora palifera* | Kenting, Taiwan |  |  |  |  |
| **Iso740** | *Isopora palifera* | Kenting, Taiwan |  |  |  |  |
